# Expectation and Evaluation of Spouse’s Filial Piety and Marital Satisfaction in China

**DOI:** 10.3389/fpsyg.2021.595854

**Published:** 2021-05-13

**Authors:** Yongxia Gui

**Affiliations:** Research Center of Psychological Health, Henan University of Economics and Law, Zhengzhou, China

**Keywords:** gender role attitude, marital satisfaction, expectation, evaluation, spouse’s filial piety

## Abstract

The present study examined the effect of expectation and evaluation of spouse’s filial piety on marital satisfaction among young Chinese couples. We administered scales assessing gender role attitude, marital satisfaction, and expectation and evaluation of spouse’s filial piety on 422 married participants and explored the relationships among these variables. The results showed the following: (1) gender role attitude mediated the relationship between participants’ gender and evaluation of their spouse’s filial piety. There was no significant gender difference in the evaluation of spouse’s filial piety; however, men were more likely to have a traditional gender role attitude, and a traditional gender role attitude leads to lower evaluation of spouse’s filial piety. Furthermore, it was found that the wife’s sibling condition influenced the participants’ expectation and evaluation of spouse’s filial piety as compared to the husband’s; (2) the evaluation of spouse’s filial piety was significantly positively correlated with marital satisfaction; and (3) women’s expectations of their husband’s filial piety moderated this relationship. The positive effects of the evaluation of spouse’s filial piety on marital satisfaction were significantly stronger when they had high expectations in this regard.

## Introduction

As a fundamental traditional virtue in China which can be traced back to thousands of years, filial piety has been criticized in the New Culture Movement (around the time of the May 4th Movement in 1919); the younger generation has now gained a lot more control over their own careers, marriages, and so on. In the meantime, social security systems have not been fully developed, and many Chinese elders still help with rearing grandchildren (Chen et al., 2011) or even live with young couples, and the lives of the two generations relied on each other’s support until now. Under this extended family background lifestyle, many researchers have reexamined the connotation of filial piety in people’s beliefs (Chen et al., 2007; Fu et al., 2016) and proposed that, instead of fully identifying with authoritarian filial piety, young Chinese people have widely accepted the flexible concept of filial piety that has emerged in recent times (that is, reciprocal filial piety) and are willing to be affectionate and dutiful toward their parents (for a detailed history of filial piety research, see Bedford and Yeh, 2019). The present research defines filial piety as the young generations’ voluntary willingness of attending to parents both physically and mentally.

Gender is always an important factor in explaining family interactions, and gender role attitude is an important psychological variable, which is both significantly related to biological sex in that men have more traditional attitudes than women (Zhang, 2006; Liu and Tong, 2014; Qiu, 2015) and more exposure-fluctuant than biological sex (Bolzendahl and Myers, 2004; Gui, 2019). As such, gender role attitude may mediate the relationship between gender and opinions about filial piety. Against a background of a long patriarchal history in China, filial piety was mostly significant for sons and daughters-in-law rather than daughters and sons-in-law. With the development of gender equality and the implementation of the only-child policy in the 1980s, nowadays daughters are more involved in supporting their parents, while daughters-in-law tend to participate less in this (Chappell and Kusch, 2007); however, culturally prescribed expectations still exert long-lasting influences in this regard. Cong and Silverstein (2008) revealed that depressive symptoms in older adults in rural China were usually reduced by assistance from daughters-in-law but sometimes increased when such support was received from sons. Brasher (2018) utilized survey data from the 2002 wave of the Chinese Survey of Family Dynamics and found that, among adult children who provide financial support to parents, women gave higher amounts of money to their in-laws than men.

Traditionally, filial piety is not only gender-related but can also be an important factor in the young couple’s marriage. In “The Book of Rites,” known as the *Liji*, which is a collection of texts describing the social forms of the Zhou dynasty, it is said: “If a son approves of his wife very much, but his parents do not like her, he should divorce her. If he does not approve of his wife, but his parents say, ‘she serves us well,’ he should behave with her in all respects as his wife, without fail, till the end of her life.” Although marriage and filial piety bind with each other so intensely as stated, only few studies have considered connecting filial piety with adult children’s romantic relationships in the present. Chen and Wu (2017) argue that there is a transmission from parent-child relationships to romantic relationships, that is, people who held strong filial piety beliefs are more likely to hold Storge and Agape loving attitudes toward their partners, which leads to satisfaction in romantic relationships; however, their research was based on undergraduate students who have not experienced actual family conflicts of interests between family members as experienced by married couples. Contrary to this “parents-partner” transmission theory, other researchers see the young couple as being responsible for negotiating various people’s interests in their intricate social network, especially each other’s parents’ interests (Huston, 2000; Milardo and Helms-Erikson, 2000). Some studies found that a daughter-in-law’s marital satisfaction was significantly influenced by her relationship with her parents-in-law (Liu et al., 2017; Cao et al., 2019) and that wives tend to feel that they are supporting their husbands by showing affection to their parents-in-law (Wong, 2000). However, no study has focused on the effect of fulfillment of spouse’s filial expectations on marital satisfaction, which is to be addressed in the present research.

The expectancy violations theory (Burgoon, 1993) predicts that falling short of an expectation (a negative violation) results in lower relational quality but that exceeding the expectation (a positive violation) is a welcome divergence linked to greater relational quality (Bachman and Guerrero, 2006), and the positive effect of ideal perception match is stronger for those with stringent ideal expectations (Campbell et al., 2001).

Marital expectation violation is expected to be significantly related to people’s marital satisfaction. This effect was proved based on personality expectations (Fletcher et al., 2000; Campbell et al., 2001; Simpson et al., 2002) and social support expectations like taking care of children (Khazan et al., 2008; Seiger and Wiese, 2011). Against the specific filial cultural background in China, some studies emphasized the importance of filial virtue in mate preference (Zhang and Kline, 2009; Jiang and Gong, 2015; Gui and Li, 2019); however, little research explores specific marital expectations about spouse’s filial piety toward one’s own parents and how these expectations influence marital satisfaction (Wu and Huang, 2012).

Based on the abovementioned theoretical and empirical evidence, the present study proposed three hypotheses based on the expectancy violations theory:

Hypothesis 1: There is a gender difference in the expectations and evaluation of spouse’s filial piety, and gender role attitude mediates the relationship between gender and expectation or evaluation of spouse’s filial piety.

Husbands may expect more from wives and evaluate wives’ filial piety behavior lower. As men tend to hold a more traditional gender role attitude, husbands set higher filial expectation standards for their wives, while wives set lower filial expectation standards for husbands.

Hypothesis 2: The evaluation of spouse’s filial piety is related to higher marital satisfaction. This implies that those who evaluate their spouses’ filial piety to their own parents higher feel more satisfied with their marriage.

Hypothesis 3: Expectations of spouse’s filial piety moderate the relationship between evaluation of spouse’s filial piety and marital satisfaction. For those who have lower expectations, the evaluation of spouse’s filial piety has a small effect on marital satisfaction, while for those who have higher expectations, evaluation of spouse’s filial piety has a large effect on marital satisfaction.

## Materials and Methods

The present study was approved by the Ethics Committee of Henan University of Economics and Law and was conducted in two phases. The first phase was conducted through a Chinese reputable online survey website to collect data. The participants had to click “I have read the consent form and agree to participate in the research” to take part in the online survey; 125 people participated, and 96 sample data were included after screening for lie-detecting items; the effective rate was 77%. In the second phase, 40 well-trained undergraduate interviewers took advantage of the spring festival vacation, which began in the end of January 2019, to conduct these interviews; they were told to contact married couples who support their parents within the Henan Province for the study sample. The volunteer participants were first asked to read the consent form and sign “consentient” for it before answering the interview questionnaire. In March 2019, when the interviewers returned to school after their vacation, 418 questionnaires were collected. After eliminating responses from some participants based on lie-detecting items and too many unanswered items, a total of 326 validated questionnaires were collected and the interviewers were paid 4 yuan per valid questionnaire; the effective rate was 78%.

Finally, data from a total of 422 questionnaires were used in the final analysis. The participants were aged 36.8 years on average (*SD* = 7.89; age was not reported by two participants), 207 were men (49.1%; gender information was missing for two participants), and all participants had at least one parent alive. Information about participants’ sibling condition (whether they were an only child or had siblings) was also collected since this was expected to influence their family role expectations. The participants had to choose one option among the following: “being the only child,” “having only sisters,” “having only brothers,” and “having both sisters and brothers.” The choice of “being the only child” was used as the reference baseline, and three dummy variables were created: “being the only child or having only sisters,” “being the only child or having only brothers,” and “being the only child or having both sisters and brothers.” Besides these, a variable for “couples’ sibling condition” reflecting the matching status of the couple in terms of their respective sibling conditions was created and used as a covariate in the following moderating effect data analysis.

### Expectation and Evaluation of Spouse’s Filial Piety Scale

Although widely explored, filial piety has never been used to assess young couples’ expectations of spouse’s filial piety. As identified in the previous research (Bedford and Yeh, 2019), also considering the adaptability to the role of child-in-law, 22 filial piety items of respecting and caring for one’s parents, attending to their needs, providing physical and financial care as they age, and memorializing them after their death were developed and administered to all participants. Two scales with corresponding items were created, namely, Expectation of Spouse’s Filial Piety and Evaluation of Spouse’s Filial Piety. For responding to each item, there were two questions. The first question was as follows: As described in this item, do you think a spouse should measure up to these criteria? The participant was asked to respond on a Likert-type scale ranging from 1 (I do not care whether he/she does this) to 5 (A spouse should definitely do this), which was treated as the expectation of spouse’s filial piety. The second question was as follows: As described in this item, how do you evaluate your spouse’s behavior toward your parents? The participant was asked to respond on a Likert-type scale from 1 (barely any) to 5 (he/she is perfect in this aspect), which was treated as the evaluation of spouse’s filial piety. Since evaluation of behavior might be more differentiated than just the expectations, we conducted exploratory and confirmatory factor analysis to extract high reliability dimensions based on the evaluation of spouse’s filial piety; then, the factor result was used for both the Evaluation and Expectation of Spouse’s Filial Piety scales since they had corresponding item descriptions.

### Gender Role Attitude Measurement

Eight gender-related items from the global program World Value Survey wave 6 (World Values Survey, 2012) were chosen as indicators of gender role attitudes. A sample item was as follows: “When jobs are scarce, men should have more right to a job than women.” The respondents rated the items on a Likert-type scale from “1” (strongly disagree) to 5 (strongly agree). A higher score on this scale indicates a traditional gender role attitude. Cronbach’s alpha was 0.652 in the present study.

### Chinese Version of the Kansas Marital Satisfaction Scale

The Chinese version of the Kansas Marital Satisfaction Scale (Shek et al., 1993; Li and Chen, 2001) was used to measure the participants’ satisfaction with their marriage. It contained items like “How satisfied are you with your marriage?” The respondents rated the items from “1” (extremely dissatisfied) to “7” (extremely satisfied). Cronbach’s alpha was 0.923 in the present research, and a higher score indicated greater satisfaction with marriage.

## Results

### Expectation and Evaluation of Spouse’s Filial Piety Scale

As noted earlier, the factor analysis results based on the evaluation of spouse’s filial piety data was used as the structure for both Expectation and Evaluation of Spouse’s Filial Piety scales. Of the total 422 questionnaires included in the final analysis, 215 were used to conduct exploratory factor analysis, and the remaining 207 were used to conduct confirmatory factor analysis. The Kaiser-Meyer-Olkin measure of sampling adequacy was 0.833, which was above the commonly recommended value of 0.600. Bartlett’s test of sphericity was also significant [χ^2^_(*df* = 66)_ = 779.703, *p* < 0.000]. Both results suggest that the correlations between different items are significant, and it is suitable to conduct a factor analysis. Exploratory factor analysis was conducted using IBM SPSS 22.0, and confirmatory factor analysis was carried out using Lisrel 8.70. Table 1 shows the results of the factor analysis of the Evaluation of Spouse’s Filial Piety scale. The analysis revealed three factors with 12 items. The first factor was “Spouse’s Devoted Filial Piety” (four items, Cronbach’s alpha 0.766); these items reflect ways of expressing filial piety in a self-sacrificing manner which costs excessive time, energy, and loyalty. The second factor was “Spouse’s Attitudinal Filial Piety” (four items, alpha 0.745); these items, with top factor loading values, reflect ways of expressing filial piety with one’s attitude, which might either be superficial or not, and the evaluation might be influenced by the observers’ attribution. The third factor was “Spouse’s Mannerly Filial Piety” (four items, alpha 0.770); these items are related to visiting parents-in-law and are relatively observable and objective, and they reflect ways of expressing filial piety with equal dignity. A higher score on this scale indicates higher evaluation of the spouse’s filial piety. The standardized regression weights of the items are given in Table 1. According to Hooper et al. (2008), we chose fit indices including the chi-square/df ratio (92.89/51 = 1.82), the RMSEA and its associated confidence interval (RMSEA = 0.063; 90% CI = 0.042–0.083), the SRMR (0.050), the CFI (0.98), and one parsimony fit index (0.61 in this research), all of which illustrate that the data fit this model well.

**TABLE 1:** Item content and factor structure of the Evaluation of Spouse’s Filial Piety scale^*a*^.

		Factors based on evaluation of spouse’s filial piety			Standardized parameter estimates
	Communality	1 Spouse’s devoted filial piety	2 Spouse’s attitudinal filial piety	3 Spouse’s mannerly filial piety	
Item 11 In leisure time, children-in-law should spend time with parents-in-law to cheer the parents up.	0.422	*0.791*^*b*^	0.003	0.112	0.78
Item 16 Children-in-law should talk with parents-in-law in order to improve their relationship	0.425	*0.714*^*b*^	−0.097	−0.076	0.80
Item 12 Children-in-law should fulfill the final wishes of the departed elderly (including parents-in-law).	0.360	*0.472*^*b*^	0.052	−0.150	0.58
Item 18 In choosing a career or decision to have kids, children-in-law should consider parents-in-law’s suggestion.	0.281	*0.330*^*b*^	0.105	−0.075	0.55
Item 7 Children-in-law should not lose temper or talk back to parents-in-law.	0.468	−0.070	*0.969*^*b*^	0.080	0.63
Item 13 Children-in-law should be patient when explaining problems to parents-in-law.	0.395	0.011	*0.539*^*b*^	−0.179	0.77
Item 8 Children-in-law should financially support parents-in-law now and then.	0.358	0.240	*0.369*^*b*^	−0.085	0.62
Item 6 Children-in-law should memorialize their ancestors (including parents-in-law) regularly on proper occasions.	0.304	0.272	*0.345*^*b*^	−0.012	0.57
Item 21 Children-in-law should visit and reunite with parents-in-law during festivals.	0.439	0.001	−0.080	−*0.790*^*b*^	0.65
Item 22 No matter how busy they are, children-in-law should make time to meet parents-in-law.	0.464	0.058	−0.025	−*0.757*^*b*^	0.69
Item 20 Children-in-law should not do dangerous things to avoid worrying their parents-in-law.	0.282	−0.019	0.184	−*0.456*^*b*^	0.60
Item 14 Children-in-law should take care of parents-in law when necessary.	0.484	0.316	0.173	−*0.367*^*b*^	0.75
Eigenvalue		4.538	1.212	1.124	
Explained variance		37.813	10.104	9.364	
Cumulative variance		37.813	47.917	57.281	

*Extraction method, principal axis factors; rotation method, Promax with Kaiser normalization. ^a,b^Rotation converged in seven iterations. The highest factor loading value for each item is written in italic, and three factors emerged based on these variables.*

The Expectation of Spouse’s Filial Piety scale with corresponding items followed this factor result, with a slight revision of questions and Likert-type choices. As shown in Table 2, Cronbach’s alpha for factor 1 “Expectation of Spouse’s Devoted Filial Piety” was 0.701, Cronbach’s alpha for factor 2 “Expectation of Spouse’s Attitudinal Filial Piety” was 0.677, and Cronbach’s alpha for factor 3 “Expectation of Spouse’s Mannerly Filial Piety” was 0.695. Both scales of Expectation and Evaluation of Spouse’s Filial Piety had high reliability and showed a correlation between different factor ratings within a reasonable range from 0.4 to 0.66.

**TABLE 2:** Descriptive statistics, correlations, and independent *t*-test between gender results of different variables^*a*^.

		F1 exp	F1 eva	F2 exp	F2 eva	F3 exp	F3 eva	Gen R atti	MS	Mean (SD)	Male	Female	*t*-value
Devoted filial piety	F1exp	1								3.7 (0.83)	3.8 (0.81)	3.7 (0.85)	1.209
	F1eva	0.658**	1	.						3.8 (0.87)	3.8 (0.87)	3.7 (0.88)	1.107
	F1exp-F1eva							0.091	−0.065	−0.05 (0.70)	−0.05 (0.71)	−0.05 (0.70)	0.024
Attitudinal filial piety	F2exp	0.605**	0.437**	1						4.2 (0.71)	4.3 (0.66)	4.2 (0.77)	1.377
	F2eva	0.438**	0.630**	0.586**	1					4.2 (0.75)	4.2 (0.75)	4.2 (0.74)	0.355
	F2exp-F2eva							0.124*	−0.169**	0.03 (0.67)	0.07 (0.67)	0.00 (0.66)	1.084
Mannerly filial piety	F3exp	0.550**	0.435**	0.618**	0.405**	1				4.4^*b*^ (0.60)	4.5^*c*^ (0.54)	4.4 (0.65)	1.410
	F3eva	0.424**	0.625**	0.451**	0.641**	0.660**	1			4.4^*b*^ (0.70)	4.4^*c*^ (0.68)	4.3 (0.71)	0.588
	F3exp-F3eva							0.116*	−0.171**	0.09 (0.54)	0.11 (0.50)	0.07 (0.58)	0.814
Gender role attitude		0.040	−0.036	−0.073	−0.180**	0.004	−0.087	1		2.7 (0.60)	2.8 (0.62)	2.6 (0.58)	2.922**
Marital satisfaction		0.260**	0.299**	0.239**	0.379**	0.184**	0.292**	−0.129**	1	5.7 (1.01)	5.9 (0.92)	5.6 (1.07)	2.714**
Cronbach’s alpha		0.701	0.766	0.677	0.745	0.695	0.770	0.652	0.923				

*p < 0.05, **p < 0.01. ^a^Evaluation scale Cronbach’s alpha was calculated based on 207 data using confirmatory data analysis, while for other scales it was based on the total 422 participants’ data (some data were occasionally excluded due to missing values), and the Cronbach’s alpha for gender role attitude was based on 415 participants’ data (ranking of the second least). ^b,c^Numbers with the same superscript indicate that they were significantly different based on paired t-test. F1, factor of spouse’s devoted filial piety; F2, factor of spouse’s attitudinal filial piety; F3, factor of spouse’s mannerly filial piety; exp and eva, expectation and evaluation of each corresponding factor; Gen R atti, gender role attitude; MS, marital satisfaction.

### Gender-Related Variables and the Expectation and Evaluation of Spouse’s Filial Piety

As shown in Table 2, the correlations among different variables were within a reasonable range, and no serious multicollinearity problem was observed; one expectation–evaluation mismatch index was calculated by subtracting the scores of evaluation from the scores of expectation for each factor. The scores of expectation and evaluation of spouses’ filial piety were all higher than the midpoint, and spouse’s devoted filial piety scored much lower than the other two dimensions.

#### Effect of Gender and Gender Role Attitude on Expectation and Evaluation of Spouse’s Filial Piety

The result of the independent samples *t*-test revealed no significant gender differences for any factor; paired *t*-test between each expectation and evaluation factor showed that, for mannerly filial piety, expectation scores were significantly higher than scores of evaluation (*t* = 3.505, *p* < 0.01), and further analysis revealed that this difference was significant only for male participants (*t* = 3.287, *p* < 0.01).

As shown in Table 2, gender differences on gender role attitude were significant (*t* = 2.922, *p* = 0.004), and there was a significantly negative correlation between gender role attitude and the evaluation of spouse’s attitudinal filial piety (−0.180).

We used bootstrap estimates and constructed a bias-corrected confidence interval (95%) to test the indirect effect of gender role attitude (Edwards and Lambert, 2007; Hayes, 2018) between gender and expectation and evaluation factor of spouse’s filial piety. The results indicated that the indirect effect of gender role attitude was significant for the evaluation of attitudinal filial piety factor (bootstrap estimate = 0.0384; bias-corrected CI: 0.0124–0.0805) and the evaluation of mannerly filial piety factor (bootstrap estimate = 0.0172; bias-corrected CI: 0.0013–0.0493). Therefore, gender role attitude mediated the effects of gender on evaluation of filial piety. Gender did not predict expectation or evaluation of spouse’s filial piety directly, but men usually display a more traditional gender role attitude (Zhang, 2006; Liu and Tong, 2014; Qiu, 2015), and a traditional gender role attitude leads to lower evaluation of spouse’s filial piety.

#### Effect of Couple’s Sibling Condition on Expectation and Evaluation of Spouse’s Filial Piety

Independent *t*-tests were conducted six times, three for the participant’s self and three for the participant’s spouse, and the result is presented in Table 3. With one exception that husbands who had only sisters evaluated their spouse’s devoted filial behavior higher than those husbands who were the only child, the other significant differences were all based on the wife’s sibling condition. For male participants, compared to the condition when their wife was the only child, husbands’ expectation and evaluation of spouse’s filial piety were higher when their wife had brothers, regardless of whether she had only brothers or both brothers and sisters. For female participants, wives who were the only child had a significantly lower expectation and evaluation of their spouses’ filial piety than wives with other sibling conditions (having brothers or sisters or both).

**TABLE 3:** Independent *t*-test comparisons of expectation and evaluation of spouse’s filial piety between different sibling conditions.

	Factor 1: devoted filial piety				Factor 2: attitudinal filial piety				Factor 3: mannerly filial piety			
Sibling conditions (*n*)	Expectation		Evaluation		Expectation		Evaluation		Expectation		Evaluation	
	Mean (SD)	*t*-value	Mean (SD)	*t*-value	Mean (SD)	*t*-value	Mean (SD)	*t*-value	Mean (SD)	*t*-value	Mean (SD)	*t*-value
**Male participants**												
1. Self is the only child (34)	3.6 (0.83)		3.6 (0.93)		4.2 (0.66)		4.1 (0.65)		4.4 (0.61)		4.3 (0.70)	
2. Self has only sisters (64)	3.8 (0.82)	1.093	4.0 (0.73)	2.007*	4.3 (0.68)	0.969	0.3 (0.74)	1.074	4.5 (0.55)	1.404	4.5 (0.59)	1.439
3. Self has only brothers (37)	3.7 (0.78)	0.260	3.6 (0.92)	0.298	4.1 (0.71)	0.077	4.0 (0.91)	0.541	4.5 (0.52)	0.940	4.3 (0.72)	0.130
4. Self has brothers and sisters (72)	3.8 (0.82)	1.346	3.8 (0.92)	1.046	4.4 (0.60)	1.524	4.3 (0.70)	1.298	4.5 (0.51)	1.290	4.4 (0.73)	0.849
1. Partner is the only child (20)	3.4 (0.76)		3.5 (0.97)		4.1 (0.53)		4.3 (0.56)		4.3 (0.55)		4.3 (0.66)	
2. Partner have only sisters (27)	3.7 (0.82)	1.205	3.7 (0.97)	0.776	4.1 (0.75)	0.026	4.0 (0.83)	1.310	4.3 (0.66)	0.201	4.2 (0.74)	0.253
3. Partner has only brothers (55)	3.9 (0.80)	2.166*	3.9 (0.82)	2.070*	4.4 (0.68)	1.559	4.2 (0.71)	0.123	4.6 (0.44)	2.673**	4.5 (0.60)	1.179
4. Partner has brothers and sisters (101)	3.8 (0.81)	1.982*	3.8 (0.85)	1.748	4.3 (0.63)	1.412	4.2 (0.79)	0.388	4.5 (0.54)	1.592	4.4 (0.72)	0.596
**Female participants**												
1. Self is the only child (35)	3.2 (0.80)		3.5 (0.89)		3.9 (0.60)		4.1 (0.66)		4.3 (0.44)		4.2 (0.58)	
2. Self has only sisters (25)	3.8 (0.90)	2.569*	4.1 (0.75)	2.895*	4.2 (0.71)	1.927	4.3 (0.55)	1.536	4.5 (0.53)	2.035*	4.6 (0.56)	2.667*
3. Self has only brothers (60)	3.7 (0.78)	2.731**	3.6 (0.94)	0.531	4.2 (0.82)	1.559	4.1 (0.89)	0.175	4.4 (0.71)	1.022	4.2 (0.85)	0.283
4. Self has brothers and sisters (92)	3.8 (0.83)	3.545**	3.8 (0.82)	2.283*	4.3 (0.78)	2.477*	4.2 (0.71)	1.329	4.4 (0.70)	1.638	4.4 (0.68)	2.262*
1. Partner is the only child (44)	3.5 (0.84)		3.6 (0.86)		4.2 (0.61)		4.2 (0.62)		4.4 (0.47)		4.3 (0.56)	
2. Partner has only sisters (55)	3.8 (0.88)	1.389	3.7 (0.91)	0.696	4.2 (0.87)	0.037	4.1 (0.84)	0.474	4.4 (0.84)	0.232	4.3 (0.84)	0.433
3. Partner has only brothers (35)	3.6 (0.90)	0.190	3.6 (0.94)	0.059	4.1 (0.88)	0.370	4.2 (0.86)	0.165	4.4 (0.66)	0.080	4.3 (0.81)	0.402
4. Partner has brothers and sisters (76)	3.7 (0.79)	1.128	3.8 (0.84)	1.355	4.2 (0.72)	0.291	4.2 (0.70)	0.044	4.4 (0.60)	0.145	4.4 (0.66)	1.386

*p < 0.05, **p < 0. 01.

**FIGURE 1:**
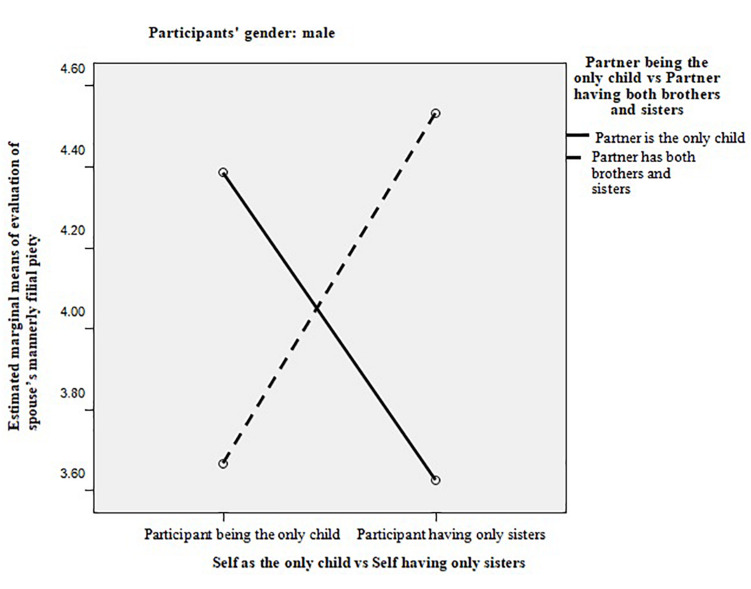
The interaction effect of couple’s sibling condition on the evaluation of mannerly filial piety for male participants.

In order to explore if there was a significant interaction effect between participants’ own and spouse’s sibling condition, nine multivariate analyses of variance with each dummy variable of sibling condition for participant’s self and participant’s spouse as the independent variable and all six factors of expectation and evaluation of spouses’ filial piety as dependent variables were conducted. The result showed that only one significant multivariate effect of interaction was found: for male participants, the interaction between “participant being the only child or having only sisters” and “partner being the only child or having siblings from both genders” was significant (Pillai’s trace = 0.178, *F* = 3.140, *p* = 0.013, partial η^2^ = 0.320). Follow-up ANOVAs were conducted to determine which dependent variable the interaction effect was significant for, and it was found that the interaction effect was significant only on the evaluation of mannerly filial piety (*F* = 6.470, *p* = 0.014, partial η^2^ = 0.126). As shown in Figure 1, if the wife was the only child, husbands with only sisters evaluated the wives’ filial piety lower than husbands who were the only child; if the wife had both brothers and sisters, husbands with only sisters evaluated their wife’s filial piety higher than husbands who were the only child.

### Relationships Between Expectation and Evaluation of Spouse’s Filial Piety and Marital Satisfaction

As shown in Table 2, the participants obtained high scores on marital satisfaction (mean = 5.7, *SD* = 1.01); men scored significantly higher than women (*t* = 2.714, *p* < 0.01), and gender role attitude was significantly negatively correlated with marital satisfaction (−0.129). Marital satisfaction was significantly correlated with both expectation and evaluation of spouse’s filial piety, and the correlation coefficient with evaluation was higher than the coefficient for expectation for each factor. It is notable that the mismatch index generated from spouse’s devoted filial piety was not correlated with marital satisfaction, while the other two were negatively correlated with marital satisfaction.

**FIGURE 2:**
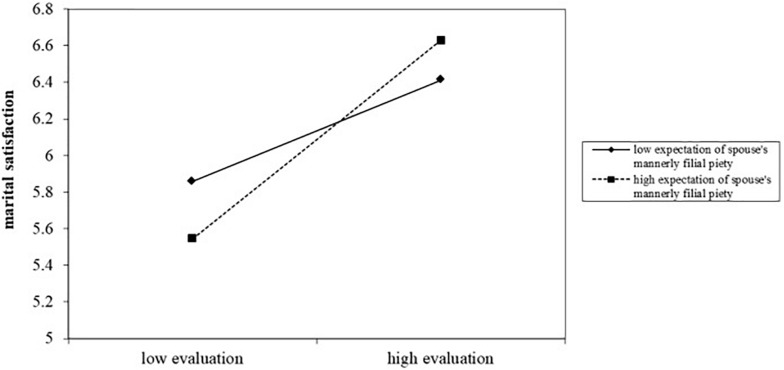
The moderating effect of expectation of mannerly filial piety on the relationship between the corresponding evaluation and marital satisfaction for female participants.

According to Hayes (2018), moderation analysis *via* PROCESS model 1 was conducted to test hypothesis 3, with each evaluation factor of spouse’s filial piety as the independent variable, the corresponding expectation factor as the moderating variable, gender role attitude and couple’s sibling condition as covariate variables, and marital satisfaction as the dependent variable. The results show that the moderation effect of expectation was only significant for the factor of evaluation of mannerly filial piety’s effect on marital satisfaction for female participants (females, *b* = 0.166, *t* = 1.999, *p* = 0.048; males, *b* = −0.074, *t* = −0.528, *p* = 0.598). With regard to data for female participants, for the low levels of filial expectation (i.e., −1 SD), filial piety evaluation was significantly related with marital satisfaction (*b* = 0.433, *t* = 3.281, *p* = 0.001), whereas for the high levels of filial expectation, filial piety evaluation was also significantly related with marital satisfaction but with a bigger slope coefficient (*b* = 0.642, *t* = 4.105, *p* < 0.001). Combined with multiple regression analysis, we plotted this significant interaction at ± 1 SD from the mean of expectation of mannerly filial piety (see Figure 2; see also Aiken and West, 1991, p. 132–134).

## Discussion

First of all, although all three factors were moderately correlated with each other, the factor of spouse’s devoted filial piety showed much lower scores than the factors of attitudinal filial piety and mannerly filial piety, and the mismatch index generated from it had no relationship with marital satisfaction, while this index showed a significant correlation in the case of the other two factors. This resonates with the dual filial piety model that filial piety has two forms, reciprocal and authoritarian, and young Chinese couples have flexible filial piety expectations from their spouses, which is closer to reciprocal filial piety (Bedford and Yeh, 2019), and the positive effect of people’s expectation of spouse’s filial piety on marital satisfaction is based more on proper filial piety behavior, that is, attitudinal and mannerly rather than devoted filial piety which is now regarded as too unrealistic and oppressive.

Although gender is closely related with family roles and men scored slightly higher than women on each factor, none of the gender differences was significant. It appears that biological sex did not influence expectation or evaluation of spouse’s filial piety directly but may have cast an influence on the evaluation of spouse’s filial piety through the mediation of gender role attitudes. This is in conflict with some studies which emphasize the role of gender on filial piety and compare gender differences in regard (Chappell and Kusch, 2007; Cong and Silverstein, 2008; Brasher, 2018). In the present study, the use of gender-neutral personal pronouns in item expression might have contributed to this small gender difference, that is, placing themselves according to gender roles in the item situations might have added to the participants’ cognitive load and cast an influence on gender comparisons. Although no significant gender difference was found directly, it appears that it was always the wives’ sibling condition which influenced the participants’ expectations and evaluation of spouse’s filial piety: male participants changed their expectation or evaluation based on their wives’ sibling condition, and so did female participants based on their own sibling condition. Marriages in the modern era, where couples perceive themselves as equals, and traditional patriarchal marriages, where males have dominance and power, exist together in China (Wang, 2016) since women’s lives have changed radically along with the development of gender equality (Quan and Gao, 2008). Daughters who are the only child have to undertake the duty of supporting their parents, while daughters who have brothers may still follow the traditional gender division of “a married-off daughter is poured out water.” The awareness of this conflict of values might contribute to the only female child’s defensive expectation that “I never expect you to support my parents, neither do you expect me to do it” for her husband. Furthermore, only men scored significantly lower on the evaluation than the expectation of spouses’ mannerly filial piety, which indicated a slight disappointment with their spouses’ mannerly filial piety, which may be attributed to the lasting effects of culturally prescribed expectations for daughters-in-law. The effects of gender role attitude and couple’s sibling condition can be explained by the social role theory, stating that people have different expectations from men and women (Chappell and Kusch, 2007; Cong and Silverstein, 2008; Brasher, 2018).

Adding to the nuclear family-focused marital expectations (Fletcher et al., 2000; Campbell et al., 2001; Simpson et al., 2002; Khazan et al., 2008; Seiger and Wiese, 2011), consistent with previous research results that filial piety could be proposed as an important virtue for mate preference (Zhang and Kline, 2009; Jiang and Gong, 2015; Gui and Li, 2019), the present study found a culture-specific marital expectation under the extended family historical background and confirmed that young Chinese couples identify with filial virtue and evaluate their spouse’s filial piety toward their parents and that a higher evaluation implies higher marital satisfaction. The present study is different from Chen and Wu (2017) focus on the transmission of filial piety belief to “Storge and Agape” love attitudes. From Chen and Wu (2017) perspective, young people interacted with parents and with romantic partners similarly; the researchers did not take into account the conflict of interest between partner and parents, and thus the study did not include a comprehensive description of family interaction in reality. Considering conflicts of interest between spouse and parents, the current study’s result confirms the expectancy violations theory that a condition which matches one’s expectations leads to a satisfactory relationship (Burgoon, 1993; Wu and Huang, 2012). Moreover, the present study reconfirmed the results of Campbell et al. (2001) that, for those with stringent expectations, the positive effect of evaluation of spouse’s mannerly filial piety on marital satisfaction was stronger, but this moderating effect was only significant for female participants.

There are some limitations of the present study. Our research is more of convenience sampling rather than representative sampling. First, online survey and face-to-face interview were both used in this study since it is difficult to collect enough volunteers’ data through the Internet. Although the two samples are comparable in effect rate and they were merged together instead of compared with each other since the online sample was quite small, there might be a difference such that the online participants have more free time and are more educated to be interested in taking part in scientific research, while the latter is composed of people of more diverse background. Unfortunately, this study did not focus enough on factors that may influence the participants’ expectations or evaluation of spouse’s filial piety, such as the area of residence, as China is a huge country with varying residential areas and varying customs, especially with a sharp contrast between urban and rural areas, or educational level since it is significantly related to gender role attitudes and neither considered other covariate’s influence such as duration of marriage. Besides these, the couple’s sibling condition may have influenced the default expectations from the marriage, but this has not been proven. The mediating mechanism of expectations from the marriage on the relationship between sibling condition and expectation or evaluation of spouse’s filial piety has also not been explored. Future research should use a more representative sample and include participants’ expectations from marriage to explore the dynamic relationships among marital satisfaction, filial expectations, and family harmony.

## Data Availability Statement

The raw data supporting the conclusions of this article will be made available by the authors, without undue reservation.

## Ethics Statement

The studies involving human participants were reviewed and approved by the Ethics Committee of Henan University of Economics and Law. The patients/participants provided their written informed consent to participate in this study.

## Author Contributions

The author confirms being the sole contributor of this work and has approved it for publication.

## Conflict of Interest

The author declares that the research was conducted in the absence of any commercial or financial relationships that could be construed as a potential conflict of interest.
